# Life‐history traits and fitness of plateau pika (*Ochotona curzoniae*) in alpine meadow ecosystem

**DOI:** 10.1002/ece3.8548

**Published:** 2022-01-25

**Authors:** Haiyan Nie, Jike Liu, Baoyang Chen

**Affiliations:** ^1^ College of Life Science and Technology Central South University of Forestry and Technology Changsha China; ^2^ College of Life Sciences Zhejiang University Hang Zhou China

**Keywords:** fitness, growth model, life‐history traits, *Ochotona curzoniae*, plateau pika

## Abstract

Plateau pika (*Ochotona curzoniae*) is an endemic mammal living in the alpine meadow ecosystem in Qinghai–Tibet Plateau. We studied life history of plateau pika by mark–recapturing method. The research results showed that fitness of plateau pika to its habitat was lower than those of many other mammal species; plateau pika adopted quick growth strategy; the life‐history features of plateau pika were accorded with Charnov (*Evolutionary Ecology Research*, 2002, *4*, 749)'s formula about life‐history classification, that is, *E*/*α* ≈ 1.35, *C*·*E* ≈ 1.7, *I*/*m* ≈ 0.3, in which *E* stands for average adult life span; α stands for age at first reproduction; *C* is reproductive effort; *I* is the size of an offspring at independence from the parent; m is average adult body mass. There does not exist dimorphism in plateau pikas. The configurations of females and males are similar; the average body mass of males is a little heavier than that of females, but the difference is not significant (*F* = 1.0854, df = 154, *p* > .3058). The juveniles exhibit a J‐form growth curve before 30‐day age and grow slower between 30 and 65‐day age and reach body mass equilibrium at about 65 days old. So, 65 days is the maturity age of plateau pika. There are 3 mortality peaks in plateau pika population. The first time is in neonate period, when only one half of juveniles can pass through this period, which implies that juvenile period is influenced strongly by natural selection; the second time is in fecundity peak period, which reflects the cost of reproduction; the third time is in the old age of plateau pika, a significant loss occurs during this period, which is the result of natural selection. The average longevity of females is longer than that of males. A female reaches the maximum life span recorded, that is, 931 days. The average longevity of all individuals is 16.33 months ≈ 490 days. The survival rate of females is higher than that of males, which reflects the cost of reproduction and society role of males. It is the outcome of natural selection. The sex ratio of neonates is 1:1; however, the sex ratio of adults is female: male = 1.31:1, which is caused by higher mortality of males over females in life history. Plateau pikas reproduced two times every year. The average gestation period of females is 18–20 days. The average litter size is 4.57 individual. The average body mass of neonates is 9.28 g. The average litter size of adult female plateau pika does not variate with age. Breeding season is between April and June. The reproductive value and fertility of 15–18 months age females are highest. The reproductive value and fertility increased with age before reproductive value and fertility peak age; however, the reproductive value and fertility decreased with age after peak age. The fitness of plateau pika (*r* = .1125) was lower than that of American pikas (*O*. *princeps*) (*r* = 2.172). The survival rate was the main factor influencing fitness. The dynamic trend of plateau pika population was coincident with *r*, that is, the plateau pika population was stable.

## Graphical abstract

Plateau pika is an endemic mammal living at the alpine meadow ecosystem in Qinghai–Tibet Plateau. We studied plateau pika life history by mark–recapturing plateau pika in the field and by tracking and recording a reproductive population in half‐captivity. The results showed plateau pika adopted quick growth strategy and supported our hypotheses that fitness of plateau pika to its habitat was lower than those of many other mammal species, etc.

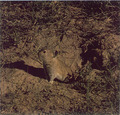

## 1 INTRODUCTION

**FIGURE 1:**
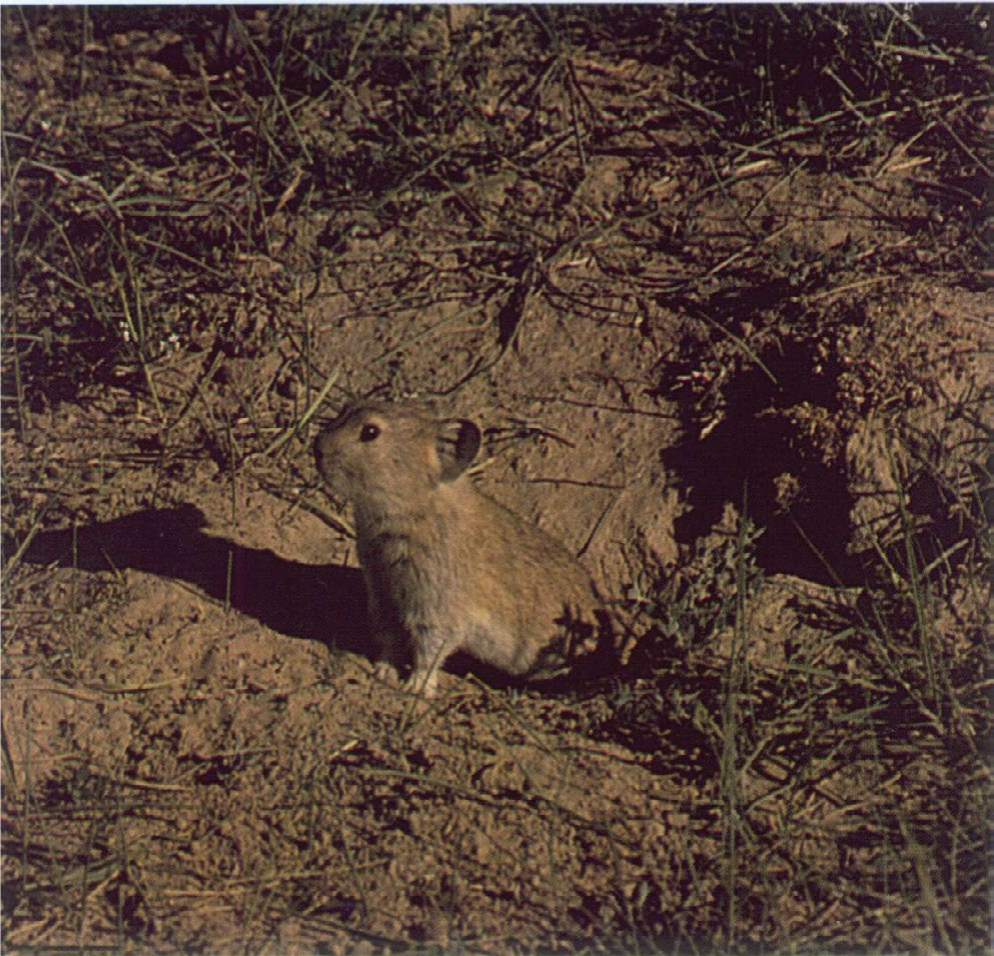
A plateau pika looking out over at the hole

Plateau pikas (*Ochotona curzoniae*) (Figure 1) are small herbivorous mammals, endemic to the Qinghai–Tibetan Plateau (Dobson et al., 2000; Smith et al., 1986; Smith & Wang, 1991; Yin et al., 2009). The roles played by plateau pikas in grassland degradation and protection are controversial (Jia et al., 2021). Plateau pikas have ever been considered a pest species on the Tibetan Plateau because they compete with livestock (yak, sheep, horses) for forage and their burrowing could contribute to soil erosion (Ekvall, 1968; Fan et al., 1999; Pech et al., 2007; Schaller, 1985; Wang et al., 1995), whereas Guo et al. (2012) supported the idea that plateau pika is a key component of alpine meadow ecosystem in the Qinghai–Tibet Plateau, and Smith and Foggin (1999), Lai and Smith (2003) held that plateau pika is a keystone species of biodiversity on the Qinghai–Tibetan Plateau. However, according to more recent research, a different opinion has been put forward illustrating that whether plateau pikas are harmful to grassland depends on population density (Sun et al., 2015; Wei et al., 2020). Population density or population dynamics correlate strongly with life‐history traits such as size at birth, growth pattern, age at maturity, and sex ratio of offspring (Brashares, 2003; Cole, 1954; Heppell et al., 2000; Stearns, 1992). Thus, life‐history traits of plateau pikas are meaningful not only to its own population development but also to the sustainability of the local ecosystem.

We mark–recaptured a wild population of plateau pika in the Haibei alpine meadow ecosystem in Qinghai–Tibet Plateau, in order to trace its life history and calculate its vital rates such as survival rate, reproduction rate, body growth rate, and fitness, and based on which analyzed its population dynamics.

## 2 METHODS

### 2.1 Study area and experimental design

The fieldwork was conducted at Haibei Alpine Meadow Ecosystem Research Station of Chinese Academy of Sciences from May 1989 to October 1991 for 3 years. The research station is located at northeast of Tibet, in a large valley oriented NW‐SE surrounded on all sides by the Qilian mountains with N latitude 37°29′–37°45′ and E longitude 101°12′–101°23′. The average altitude of mountain area is 4,000 m above sea level and 2,900–3,500 m for valley area. The Datong River passes south of the area. The landscape is characterized by large mountain ranges with steep valleys and gorges interspersed with wide intermountain grassland basins. The natural conditions, vegetation, and soil structure of study area had been reported (Xia, 1982).

The avian and mammalian predators observed in the study area were the falcon *Falca tinnunculus*, the buzzard *Bulteo hemilasius*, the weasels *Mustela altaica* and *M*. *eversmanni*, the wolf *Canis lupus*, and the foxes *Vulpes vulpes* and *V*. *ferrilatus*. The dominant species among them were the buzzard, the falcon, and the weasels (Liu et al., 1982).

The experimental site was located at alpine meadow area dominated by *Kobresia humilis*, which was the natural habitat for plateau pika. The experimental site was enclosed with 0.5 × 0.5 cm iron wire netting to keep the vegetation in the site undisturbed by grazing of domestic animals or movement of local residents. Area of the experimental site was 4 ha (200 × 200 m). Live‐trapping stations were staked outside burrows being used by plateau pikas. Whether the burrows were utilized or abandoned by plateau pikas was judged according to active burrow‐judging method (Liu et al., 1980).

Five small enclosures were set up with dense‐meshed iron‐wire‐netting near the large mark–recapture experimental enclosure, each with an area of 0.04 ha (20 × 20 m). At the back of each small enclosure, a hole was dug underground, 1.50 m below the surface. A 0.3 × 0.3 × 0.3 m wooden box was fixed in each hole for rearing pikas. The box was connected with the small enclosure above by a wire‐netting tube. The animals reared in the box could climb to the enclosure for foraging or resting through the tube.

In the reproductive season, live‐trapped pregnant females were put into the wooden boxes and reared in it, fed daily with natural food of plateau pikas, such as *Kobresia humilis*. The pregnant females were observed every day from April to October, and the delivery time of each female as well as the sex, body mass of every neonate was recorded. The neonates were weighed daily and the body mass and age of each neonate were recorded daily.

The individuals born in the rearing box and those captured in the wild lived in the same environment, or in other words, they lived under the same selective pressure, so their life‐history features should be similar. Therefore, the corresponding relationship between the age and body mass of individuals born in the rearing box could be applied to that of the individuals captured in the field, according to which we could estimate the age of the individual captured in the field.

### 2.2 Data collection and statistical analyses

#### 2.2.1 Population density

There were seven trapping days in the middle period of every month. At dusk of the day before a trapping session, the traps were set open, bedded with clean cotton, and baited with fresh carrots. The cages were examined 1–3 times each day within a trapping session and covered with hardboards of 2 cm thickness for reducing trap deaths caused by high temperature. Upon first capture, each animal was marked with a numbered aluminum ear tag. The recaptured individuals were released at the point of capture after the following data were recorded on standard card: tag number, sex, body mass, station number, reproductive condition, and general condition.

Population density for every enclosure within each trapping session was estimated separately using the jackknife estimator for model Mh (including heterogeneity in capture rates) in the program CAPTURE (Nie & Liu, 2005; Otis et al., 1978).

#### 2.2.2 Estimation of important life‐history characteristics

Important life‐history parameters were calculated according to data recorded on mark–recapture card and reproductive data obtained from reproductive populations living in the underground boxes.

The dynamic life table of plateau pika population was constructed based on mark–recapture data of individuals which were juveniles while caught at the first time, that is, the ages of the captured individuals could be estimated, and the fates of these juveniles were traced all lifelong.

The life table consisted of the following statistic parameters: (1) age‐specific mortality rate; (2) age‐specific survival rate; (3) age‐specific life expectancy; (4) life expectancy at maturity; (5) age‐specific reproductive value (*v_x_*); (6) age of maturity (*α*). All these parameters could be calculated directly from original data on mark–recapture card except *V_x_*. The formula for calculating *V_x_* is: $V_{x}=\sum_{t=x}^{\omega }\left(\frac{l_{t}}{l_{x}}\right)\cdot m_{x}=m_{x}+\sum_{t=x+1}^{\omega }\left(\frac{l_{t}}{l_{x}}\right)\cdot m_{t}$, in which *V_x_* stands for reproductive value of females of age *x*, *m_t_* stands for age‐specific fecundity of age *t*, *t* is the age older than age *x*, *ω* is the maximum reproductive age, *l_t_* is the survival rate of females of age *t*, *m_t_* is the fertility of females of age *t*. *V_x_* can be divided into two parts, present fertility and future fertility expectancy, the latter is called residual reproductive value (Williams, 1966). *V_x_* reflects the average contribution of females of age *x* can do for the future increase of population (Pianka, 1978).

The following parameters could be calculated from life table: (1) juvenile mortality (*Z*); (2) adult mortality (*M*); (3) subadult mortality (*M_s_*); (4) annual fecundity (*b*); (5) survival rate of females to the age of first reproduction (*S*), or average chance of breeding, $S=l_{x}=e^{‐\int_{0}^{\alpha }Z\left(x\right)dx}=e^{‐\bar{Z}\cdot \alpha }$, in which *l_x_* is survival rate to age *x*, *α* is the age of first reproduction, *Z*(*x*) is the instant mortality of age i, $\bar{Z}$ is the average of instant mortalities of juveniles; (6) average adult life span (*E*), $E=\int_{\alpha }^{\infty }\frac{l_{x}}{s}dx$ **;** (7) average reproductive effort (*C*), *C* is average mass (per adult) devoted to reproduction per unit of time, divided by the average adult body mass (*m*):

$$C=\frac{R}{m}=\frac{\int_{\alpha }^{\infty }l_{x}\cdot R_{x}dx/\int_{\alpha }^{\infty }l_{x}dx}{\int_{\alpha }^{\infty }l_{x}\cdot m_{x}dx/\int_{\alpha }^{\infty }l_{x}dx}=\frac{\int_{\alpha }^{\infty }l_{x}\cdot m_{x}\cdot C_{x}dx}{\int_{\alpha }^{\infty }l_{x}\cdot m_{x}dx}$$

in which *R* is average reproductive allocation per unit of time, *m* is average adult body mass, *R_x_* is adult reproductive allocation at age *x* per unit of time, *m_x_* is body size at age *x*, *C_x_* is the adult reproductive effort at age *x* per unit of body mass, *R_x_* = *C_x_* · *m_x_* (Charnov, 2002); (8) net reproductive rate (*R*_0_): the average number of daughters produced over a mother's lifetime. Since here we wish to count total offspring produced, we simply assume a 1:1 primary sex ratio and multiply *R*_0_ by 2. If *I* stands for the size (mass) of an offspring at independence from the parent, *b_x_* stands for the number of daughters produced by an age *x* female, then

$$\begin{array}{c} 2\cdot R_{0}2\int_{\alpha }^{\infty }l_{x}\cdot b_{x}dx=\int_{\alpha }^{\infty }\frac{l_{x}\cdot R_{x}}{I}dx=\frac{S}{I}\int_{\alpha }^{\infty }\frac{l_{x}\cdot C_{x}\cdot m_{x}}{S}dx\\ =S\cdot \left[\frac{\int_{\alpha }^{\infty }\frac{l_{x}}{S}dx}{1}\right]\cdot \left[\frac{\int_{\alpha }^{\infty }\frac{l_{x}\cdot C_{x}\cdot m_{x}}{S}dx}{\int_{\alpha }^{\infty }\frac{l_{x}}{S}\cdot m_{x}dx}\right]\cdot \left[\frac{1}{I}\right]\cdot \left[\frac{\int_{\alpha }^{\infty }\frac{l_{x}\cdot m_{x}}{S}dx}{\int_{\alpha }^{\infty }\frac{l_{x}}{S}dx}\right] \end{array}$$

i.e, $2\cdot R_{0}=e^{‐\bar{Z}\cdot \alpha }\cdot \left[C\cdot E\right]\cdot \left[\frac{m}{I}\right]\Rightarrow \left[C\cdot E\right]=\frac{2R_{0}}{e^{‐\bar{Z}\cdot \alpha }}\cdot \left[\frac{m}{I}\right]=2R_{0}/S\cdot \frac{m}{I}$

Survival rate of each month was calculated according to the following formula: $\hat{S}$ = (*B*_2_ · *R*_13_)/[*B*_1_ · (*R*_23_ − 1)], in which *Ŝ* was survival rate or survival estimation, *B*_1_ was the quantity of individuals captured in trapping session I, *B*_2_ was the quantity of individuals captured in trapping session II, *R*_13_ was the quantity of individuals marked in trapping session I and recaptured in trapping session III, *R*_23_ was the quantity of individuals marked in trapping session II and recaptured in trapping session III.

#### 2.2.3 Body growth model

We substituted the body growth data of individuals in half‐captivity (neonates born in the underground rearing box) for *m* in the following simulation model: d*m*/dt = a•*m* ^0.75^ −*b*•*m* (Charnov et al., 2001). Parameters *a* and *b* were estimated according to experimental data, and precision of the model was tested.

#### 2.2.4 Fitness index

Fitness of mammal species can be determined by examining mortality pattern and reproduction pattern of the species (Gaillard et al., 2000; Promislow & Harvey, 1990; Stearn, 1976, 1992). If *f* stands for population fertility, *m* stands for mortality, then the intrinsic increase rate of population *r* = *f* − *m*, that is, *r* is an index composed of population fertility and mortality and can be used as fitness index of population (Fisher, 1930; Schaffer, 1974; Stearns, 1992; Williams, 1966).

Sex ratio of almost all mammal species is close to 1:1 (Clutton‐Brock & Iason, 1986). Suppose the initial population density is *N*, adult mortality is *M*, juvenile mortality is *Z*(/Mon), according to the definition of intrinsic increase rate of population, then

$$\begin{array}{c} r=\frac{N\times b‐N\times b\times \left(Z+Z^{2}+\cdots +Z^{12}\right)‐N\times \left(M+M^{2}+\cdots +M^{12}\right)}{N}\\ =\frac{(1‐2Z+Z^{13})\cdot b}{1‐Z}‐\frac{(1‐M^{12})\cdot M}{1‐M} \end{array}$$

#### 2.2.5 Life‐history tempo

The ratio of fertility rate to age at first reproduction *T* = *F*/*α* was used to discriminate life‐history tempo of mammals. If *T* > 0.60, the species belongs to “fast” mammals, which were characterized by early maturity, short life spans, low survival rates, and high fertility and projected population growth rate (*λ*) compared to “slow” (*T* < 0.15) mammals (Oli, 2004).

## 3 RESULTS

### 3.1 Mortality pattern, life expectancy, and life span

Mortality rates of plateau pikas varied with age, though the variation range of males was not as wide as that of females. The mortality rates of males were rather high at almost all periods, but there were 3 mortality peaks in females' lifetime. The first peak was in neonate period, in which only one half of juveniles could pass through; the second peak was in breeding period; the third peak was in the old age of plateau pikas, a significant loss occurred during this period (Tables 1 and 2).

**TABLE 1:** Dynamic life table of male plateau pika (*O*. *curzoniae*)^a^

Age (*x*)	Total Alive	Survival Rate (*l_x_*)	Age‐Specific Mortality (*d_x_*)	Age‐Specific Mortality Rate (*q_x_*)	Life Expectancy (*e_x_*)
0–3	228	1.0000	112	0.4912	1.8805
3–6	116	0.5088	65	0.5604	2.0168
6–9	51	0.2237	16	0.3137	2.2694
9–12	35	0.1535	13	0.3714	2.0672
12–15	22	0.0483	11	0.5000	2.0043
15–18	11	0.0219	6	0.5455	2.4028
18–21	5	0.0132	2	0.4000	1.7019
21–24	3	0.0088	1	0.3333	1.5016
24–27	2	0.0044	1	0.5000	1.0029
27–30	1		–	–	0.5000

^a^ *x* = specific age (month); *l_x_* is age‐specific survival rate; *d_x_* is the number of individuals die in age *x* → *x* + 1; *q_x_* is the mortality in age *x* → *x* + 1; *e_x_* is life expectancy of age *x*.

**TABLE 2:** Dynamic life table of female plateau pika (*O*. *curzoniae*)

Age (*x*)	Total alive	Survival rate (*l_x_*)	Age‐specific mortality (*d_x_*)	Age‐specific mortality rate (*q_x_*)
0–3	353	1.0000	182	0.5156
3–6	171	0.4844	98	0.5731
6–9	73	0.2068	18	0.2466
9–12	55	0.1558	19	0.3455
12–15	36	0.1020	18	0.5000
15–18	18	0.0510	10	0.5556
18–21	8	0.0227	2	0.2500
21–24	6	0.0170	1	0.1667
24–27	5	0.0142	3	0.6000
27–30	2	0.0057	1	0.5000
30–33	1	0.0028	–	–

The average mortality rates of males (${\bar{q}}_{m}$ = 0.425 ± 0.12) were higher than that of females (${\bar{q}}_{f}$ = 0.388 ± 0.15). Correspondingly, the average survival rate of adult females was higher than that of adult males (Table 3). The average survival rate of adults was higher than that of juveniles (Tables 3 and 4).

**TABLE 3:** Monthly survival rate estimation of adults

Year	Month	Male					Female				
		*B*_1_	*R*_13_	*B*_2_	*R*_23_	*Ŝ*	*B*_1_	*R*_13_	*B*_2_	*R*_23_	*Ŝ*
1989	April	10	5	85	79	0.5449	10	7	88	82	0.7080
	May	85	42	8	7	0.6588	88	69	27	27	0.7356
	June	8	5	16	14	0.7692	27	22	35	34	0.8642
	July	16	6	21	13	0.6563	35	15	9	7	0.6429
	Aug.	21	6	15	9	0.5357	9	4	13	11	0.5778
	Sept.	15	4	5	4	0.4444	13	5	7	6	0.5385
	Oct.	5	2	14	11	0.5600	7	3	13	10	0.6190
1990	April	14	7	32	29	0.5714	13	8	40	37	0.6838
	May	32	14	9	7	0.6563	40	30	17	17	0.7969
	June	9	5	5	5	0.6944	17	12	7	7	0.8235
	July	5	3	6	10	0.4000	7	4	6	6	0.6857
	Aug.	10	3	6	6	0.3600	6	3	7	7	0.5833
	Sept.	6	2	3	4	0.3333	7	3	5	5	0.5357
	Oct.	3	1	18	13	0.5000	5	3	31	30	0.6276
1991	April	18	8	20	17	0.5555	31	21	23	23	0.7082
	May	20	9	8	7	0.6000	23	16	18	18	0.7366
	June	8	4	5	5	0.6250	18	14	18	18	0.8235
	July	5	2	9	7	0.6000	18	10	7	7	0.6481
	Aug.	9	3	7	6	0.4600	7	2	9	6	0.5143
		$\Sigma \hat{S}$ = 11.0851, $\bar{s}$ = 0.5834					$\Sigma \hat{S}$ = 12.8524, $\bar{s}$ = 0.6764				

**TABLE 4:** Monthly survival rate estimation of juveniles

Year	Month	Male					Female				
		*B*_1_	*R*_13_	*B*_2_	*R*_23_	*Ŝ*	*B*_1_	*R*_13_	*B*_2_	*R*_23_	*Ŝ*
1989	April	8	4	53	52	0.5196	13	6	60	57	0.5769
	May	53	35	21	21	0.5943	60	33	13	12	0.6500
	June	21	9	6	5	0.6429	13	5	8	5	0.7692
	July	6	2	14	14	0.3589	8	3	31	29	0.4152
1990	April	14	6	92	88	0.4532	22	10	110	102	0.4950
	May	60	26	14	11	0.5833	70	34	39	31	0.6314
	June	5	3	10	11	0.6000	22	12	9	8	0.7013
	July	6	3	20	30	0.4482	2	1	39	39	0.5132
1991	April	20	9	45	42	0.4821	32	17	110	109	0.5361
	May	25	12	18	16	0.5760	87	39	40	29	0.6404
	June	10	5	6	6	0.6060	22	8	30	20	0.6459
		$\Sigma \hat{S}$ = 5.8842, $\bar{s}$=0.5349					$\Sigma \hat{S}$ = 6.8915, $\bar{s}$ = 0.5824				

Monthly survival rate of adults variated evidently: The highest survival rate was in June, and the lowest was in September (Table 3). The survival pattern of female juveniles was similar to that of male juveniles (Table 4).

The life expectancy of female neonates (6.33 month) was longer than that of male neonates (5.64 month). The life expectancy of all neonates was 6 months. The life expectancy of females at maturity (6.87 month) was longer than that of males at maturity (6 month) (Tables 5 and 6).

**TABLE 5:** Life expectancy estimation of female plateau pika

Age (month) (*x*)	Known alive (*l_x_*)	Mortality (*d_x_*)	Mortality rate (*q_x_*)	Average months lived (*L_x_*)	Total months lived (*T_x_*)	Life expectancy (*e_x_*)
0–3	1,000.00	512.82	0.513	782.46	2,113.69	2.11
3–6	487.18	139.56	0.286	417.40	1,331.23	3.17
6–9	347.62	85.72	0.247	304.76	913.83	2.63
9–12	261.90	90.47	0.345	216.67	609.07	2.33
12–15	171.43	85.72	0.500	128.57	392.40	2.29
15–18	85.71	16.74	0.195	77.34	263.83	3.08
18–21	68.97	5.14	0.075	66.40	186.39	2.70
21–24	63.83	10.64	0.167	58.51	120.09	1.88
24–27	53.19	31.91	0.599	37.24	61.58	1.16
27–30	21.28	7.58	0.356	17.49	24.34	1.14
30–33	13.70	13.70	1.000	6.85	6.85	0.50
∑	2,574.81	1,000.00		2,113.69	6023.4	

**TABLE 6:** Life expectancy estimation of male plateau pika

Age (month) (*x*)	Known alive (*l_x_*)	Mortality (*d_x_*)	Mortality rate (*q_x_*)	Average months lived (*L_x_*)	Total months lived (*T_x_*)	Life expectancy (*e_x_*)
0–3	1,000.00	467.89	0.468	798.72	1,875.45	1.88
3–6	532.11	237.31	0.446	408.96	1,076.73	2.73
6–9	294.86	92.49	0.370	248.56	667.77	2.27
9–12	202.31	75.14	0.371	164.74	419.21	2.07
12–15	127.17	61.30	0.482	96.52	254.47	2.00
15–18	65.87	9.05	0.137	61.35	157.95	2.40
18–21	56.82	22.73	0.400	45.46	96.60	1.70
21–24	34.09	11.36	0.333	28.41	51.14	1.50
24–27	22.73	11.37	0.500	17.05	22.73	1.00
27–30	16.36	16.36	1.000	5.68	5.60	0.50
∑	2,352.20	1,000.0		1,875.45	4,627.73	

The average longevity of females (17.30 ± 0.71 month) was longer than that of males (15.43 ± 0.52 month). The longest life span was recorded by a female (931 days ≈ 2.55 year). The average longevity of all individuals was 490(±19.2) days ≈ 16.33 months.

### 3.2 Reproductive features of plateau pika

Most individuals reproduced during April–June (96%) and gave birth twice every year, only few individuals bred in July and August (4%). It was difficult to judge the exact first reproduction age for live‐trapped adult females; therefore, only the reproduction data of females which were trapped as juveniles for the first time and traced lifetime long were used in the reproduction analysis. There were 41 juvenile females captured, traced lifetime long, and their reproduction data were recorded. All the juvenile females did not reproduce after maturation in the year they were born. They all bred in April, May, or June in the second year. The average age of first reproduction was 354.34 ± 30.65 (day), *n* = 41 or 12 ± 1.02 (month), *n* = 41 (Figure 1).

**FIGURE 2:**
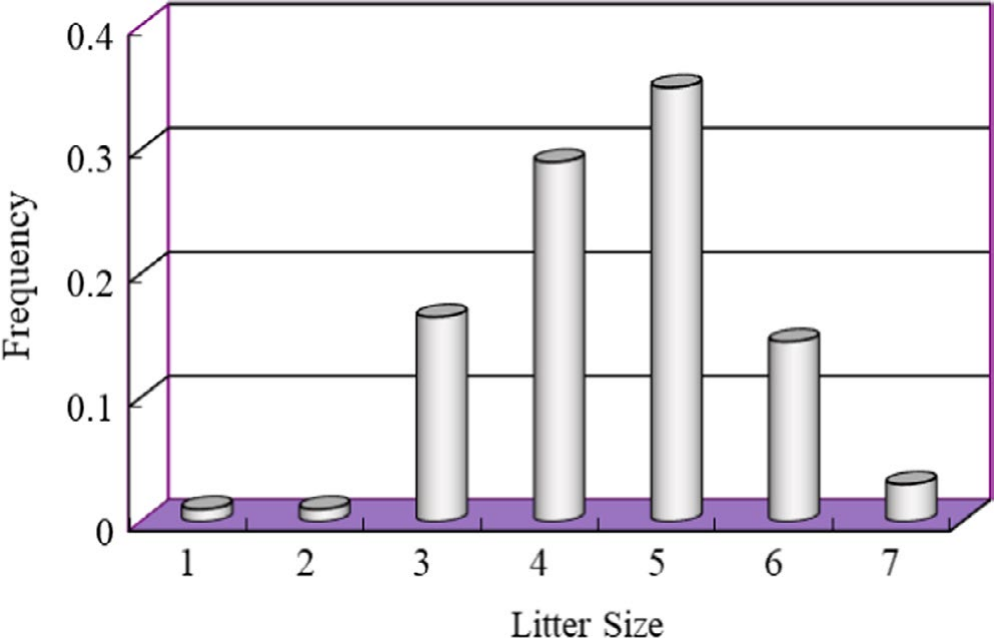
Distribution of Litter Size Frequency

The litter size range was between 1–7 individuals with average litter size 4.61 ± 0.13. The size of most litters was 4–5 individuals, and the second most was 3 or 6 individuals; occasionally, the litter size could reach 7 individuals (Figure 2).

The average gestation period was 18–20 days. The average litter size was 4.57 individuals. The average body mass of neonates was 9.28 g. The average litter size did not vary with age of mother plateau pikas (Table 7).

**TABLE 7:** General life‐history traits of plateau pika

Life‐history Traits	Female			Male			Male + female		
	Average	range of variation	sample number	Average	range of variation	sample number	Average	range of variation	sample number
First‐reproduction Age (month)	12	8–13	41						
Weight of Neonates (g)	9.28	8–11	12	9.28	8–11	12	9.28	8–11	24
Weight of Adults (g)	149.17	68–238	140	152.49	107–250	147	149.27	68–250	287
Weaning Weight (g)	19.75	17–24	8	20.66	18–24	6	19.75	17–24	14
Average Weight of Juveniles at Independent Age (g)	53.09	48–60	21	49.3	54–62	10	49.98	48–62	31
Adult Life Span (month)	16.20	2.17–31		15.50	2.17–30				

The reproductive value varied with age (Table 9). The peak age of reproductive value and fertility was 15–18 months. The reproductive value and fertility increased with age before the peak age but decreased with age after peak age.

Female fertility of 21‐ to 24‐month‐old individuals was the highest, and fertility of 12–15, 15‐ to 18‐month‐old individuals was the second highest, female fertility of 0–9 and 18‐ to 21‐month‐old individuals was the lowest (Table 8). The reproductive value of 30–33 months old individuals was 0, which meant individuals of this age had stopped breeding. The reproductive value of 15–18‐month‐old females was highest, which meant the contribution of this age period to the future generation was the highest. The reproductive value of 12‐ to 15‐month‐old females was the second highest (Table 9). The residual reproductive value of 18‐ to 21‐month‐old females was the highest (Table 9).

**TABLE 8:** Female fertility estimation^a^

Age (month)	Female number (*n_x_*)	Pregnant females (*B_x_*)	Average litter size	Total offspring (∑*n*)	Female offspring	Fertility (*m_x_*)
0–3	353	0	0	0	0	0
3–6	171	0	0	0	0	0
6–9	73	0	0	0	0	0
9–12	55	41	4.83	198.03	99.02	1.800
12–15	36	33	5.27	173.91	86.96	2.415
15–18	18	17	5.16	87.72	43.86	2.437
18–21	8	0	0	0	0	0
21–24	6	6	5.29	31.74	15.87	2.645
24–27	5	4	5.08	20.32	10.16	2.032
27–30	2	1	5.00	5.00	2.50	1.250
30–33	1	0	0	0	0	0

^a^ Fertility *m_x_* = age‐specific female offspring born per adult female, *m_x_* = ∑*n*/2 *n_x_* according to sex ratio (1:1.04) from semi‐artificial feeding box. Average fertility *m* = 1.972.

**TABLE 9:** Age‐specific reproductive value (*V_x_*) of plateau pika (*O*. *curzoniae*)

Age (month)	Pivotal Age (*x*)^*^	Age structure (*n_x_*)	Survival Series (*l_x_*)	Fertility (*m_x_*)	*l_x_*.·*m_x_*	*x*·*l_x_* ·*m_x_*	Reproductive Value (*v_x_*)
0–3	1.5	353	1,000	0	0	0	1.398
3–6	4.5	171	487.18	0	0	0	2.872
6–9	7.5	73	347.62	0	0	0	4.023
9–12	12	55	261.90	1.800	471.42	5657.04	5.341
12–15	14	36	171.43	2.415	414.00	5796.05	5.369
15–18	16	18	85.71	2.437	208.88	3342.00	6.064
18–21	20	8	68.97	0	0	0	4.411
21–24	24	6	63.83	2.645	168.83	4051.93	4.766
24–27	26	5	53.19	2.032	108.08	2,810.13	2.545
27–30	29	2	21.28	1.250	27.288	791.34	1.282
30–33	31.5	1	17.70	0	0	0	0

^*^Pivotal age is the representative age in an age‐specific period.

### 3.3 Body growth model

Average body mass of males (152.49 g ± 1.59) was slightly heavier than that of females (149.17 g ± 1.32), but the difference was not significant (*F* = 1.0854, df = 154, *p* > .3058).

**FIGURE 3:**
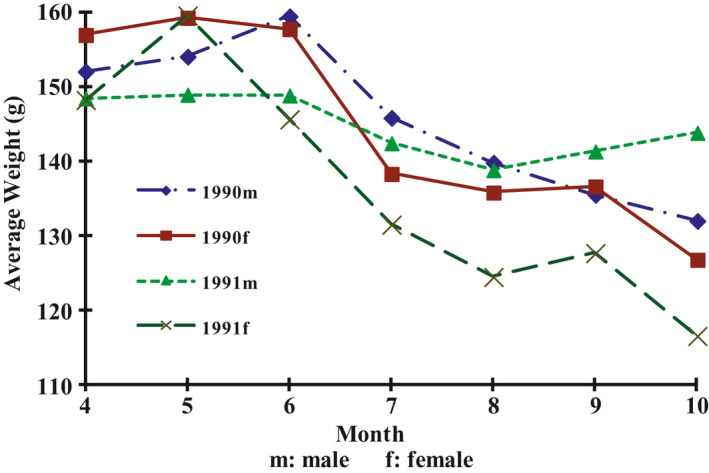
Monthly Variation of Adult Average Weight

**FIGURE 4:**
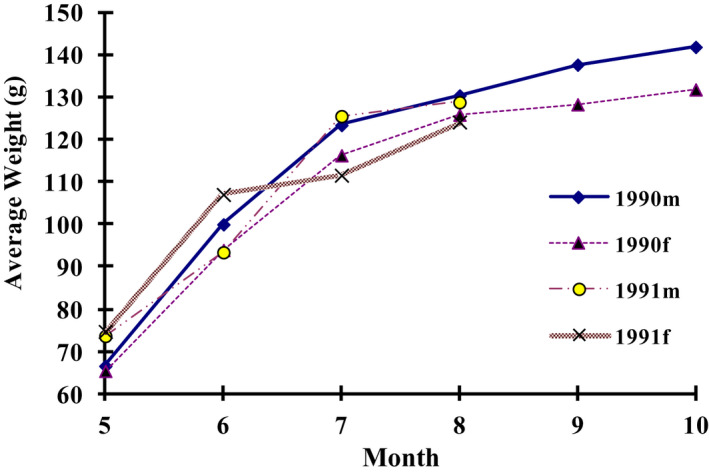
Monthly Variation of Juvenile Average Weight

Body mass of males and females decreased monthly (Figure 3). Both the average body mass of males and females were high in the breeding season, that is, from April to June, and decreased monthly from July to October. In contrast, the average body mass of juveniles increased monthly (Figure 4).

**FIGURE 5:**
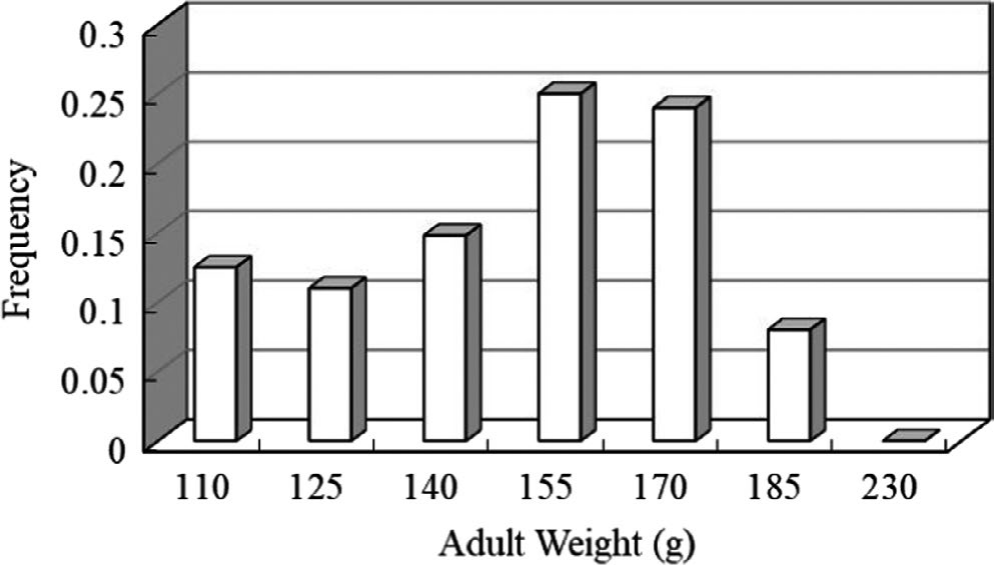
Weight distribution of adults

The minimum adult body mass was 110 g, and the maximum body mass was 230 g, with average body mass 149.27 g ± 3.41. The curve of body mass distribution was approximately normal distribution. Most individuals weighted between 155–170 g and 170–185 g, the heaviest and lightest individuals were fewer (Figure 5).

**FIGURE 6:**
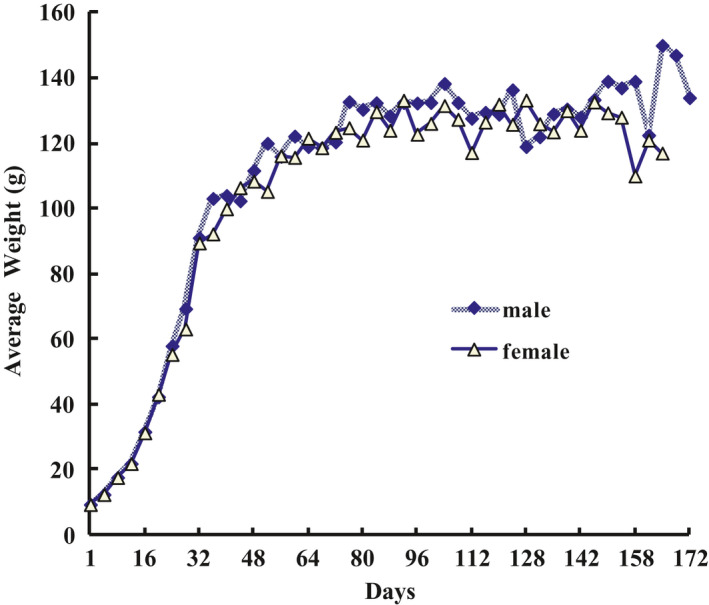
Daily Growth of Juvenile Average Weight

Juveniles weaned at the age of 10 days and became independent at the age of 30 days. The juveniles grew quickly before 30‐day age, exhibiting a J‐form growth curve, and then grew more slowly between 30–65‐day age, and reached body mass equilibrium at about 65 days old (Figure 6). The body growth of plateau pikas could be divided into four stages: (1) 1–10 days age, neonate period, in which milk was the only food; (2) l0‐30 days age, juvenile period, which was the fast growth period; (3) 30–65 days age, subadult period, in which the body growth slowed down evidently; (4) after 65 days age, adults, when body growth practically stopped.

No dimorphism existed in plateau pikas. The contours of females and males were similar; the average body mass of males was a little heavier than that of females, but the difference was not significant (*F* = 1.0854, df = 154, *p* > .3058, one‐tail). Thus, the body mass data of males and females could be merged together to form a simulation model of body growth. Substituting body mass data for parameter *m* in the body growth model for mammals (Charnov et al., 2001); a simulation model was constructed according to Lagrange median theorem based on the body mass data of plateau pikas:

(1) $$\frac{\mathrm{dm}}{\mathrm{dt}}=6.5266\cdot m^{0.75}‐12.1787\cdot m$$

in which, *m* stood for average body mass, *t* was the age, and parameters a was a constant determined by taxon, b was a metabolism constant of cells, which were calculated with Excel statistic software in PC computer as $\hat{a}$ = 6.5266; $\hat{b}$ = 12.1787. The integral formula for this model was:

$$m={\left(\frac{a}{b}+\frac{1}{b}\cdot e^{‐\frac{b}{4}t}\right)}^{4}={\left(0.5359+0.0821e^{‐3.0447t}\right)}^{4}$$

The average precision of this model was 95.5%.

### 3.4 The fitness level of plateau pikas among mammalians

A life‐history feature table and a life‐history strategy analysis table of 65 mammal species belonging to 9 orders have been compiled according to their life table data, among which the fitness indexes of 46 species have been calculated (Table 10). The fitness index of plateau pikas was ranked at the 43rd position, only higher than those of bank vole (*Clethrionmys glareolus*), chimpanzee (*Pan troglodytes*), and African elephant (*Loxodonta africana*). The fitness index of plateau pikas (*r* = .1125, Table 10) was much lower than that of American pikas (*O*. *princeps*) (*r* = 2.172).

**TABLE 10:** Fitness index of mammals

Species	Reproduction parameter				Survival parameter			Fitness index
	LS	Lits/y	*b*	*δ*	*S_α_*	*Z*	*M*	*r*
**Lagomorpha**								
**Ochotonidae**								
*Ochotona princeps*	2.73	2.00	2.73	0.357	0.210	0.157	0.047	2.1723
*O. curzoniae*	4.57	2.00	2.10	0.285	0.156	0.418	0.324	0.1125
**Leporidae**								
*Lepus europaeus*	2.46	3.99	4.91	0.155	0.423	0.108	0.058	4.2540
*Oryctolagus cuniculus*	4.74	5.09	12.10	0.135	●	●	0.283	●
*Sylvilagus floridanus*	3.86	4.58	8.84	0.098	0.425	0.202	0.128	6.4550
**Rodentia**								
*Myocasteridae*								
*Myocaster coypus*	4.54	2.76	6.26	0.208	0.273	0.154	0.079	5.0340
**Sciuridae**								
*Spermophilus armatus*	4.99	0.89	2.22	0.280	0.288	0.104	0.055	1.9043
*S. beldingi*	5.00	0.82	2.05	0.300	0.284	0.105	0.054	1.7525
*S. dauricus*	●	0.82	●	●	0.328	0.093	0.052	●
*S. lateralis*	5.15	0.82	2.11	0.421	0.156	0.108	0.050	1.8023
*Marmota flaviventris*	4.15	0.82	1.70	●	0.161	0.051	0.033	1.5752
*Tamias striatus*	4.14	1.75	3.62	0.300	0.359	0.140	0.110	2.9071
*Sciurus carolinensis*	2.85	1.67	2.38	0.229	0.316	0.115	0.049	1.5562
*Tamiasciurus hudsonicus*	3.82	0.82	1.57	0.299	0.291	0.091	0.057	1.3522
**Zapodidae**								
*Zapus hudsonius*	5.40	0.82	2.21	0.422	0.110	0.184	●	●
*Z. princeps*	4.46	0.70	1.56	●	0.301	0.076	0.052	1.3770
**Muridae**								
*Apodemus flavicollis*	5.96	2.88	8.59	0.325	0.415	0.301	0.303	4.4560
**Cricetidae**								
*Clethrionmys glareolus*	4.86	3.39	8.24	0.450	0.390	0.494	0.274	−0.1804
*Peromyscus leucopus*	4.89	●	●	0.471	●	●	0.440	●
*P. maniculatus*	4.28	4.67	9.99	0.389	0.407	0.391	0.316	3.1140
**Primates**								
*Macaca Sinica*	1.00	0.69	0.34	●	0.168	0.031	0.005	0.3240
*Pan troglodytes*	1.05	0.19	0.10	0.247	0.384	0.007	0.006	0.0933
**Insectivora**								
**Talpidae**								
*Talpa europaea*	4.10	0.82	1.68	●	0.494	0.059	0.061	1.5097
*Tachyoryctes Splendens*	1.50	2.11	1.59	●	0.523	0.124	0.045	1.3181
**Erinaceidae**								
*Erinaceus europaeus*	4.47	1.73	3.87	0.180	●	●	0.033	●
**Carnivora**								
**Ailuropodidae**								
*Ailuropoda Melanoleuca*	1.56	0.70	0.55	0.083	0.394	0.024	0.020	0.5161
**Ursidae**								
*Ursus americanus*	2.30	0.42	0.49	0.271	●	●	0.015	●
**Phocidae**								
*Phoca hispida*	1.00	0.80	0.40	0.149	0.234	0.029	0.008	0.3801
*Halichoerus grypus*	1.00	0.85	0.43	0.255	0.290	0.021	0.012	0.4086
*Mirounga leonina*	1.00	0.86	0.43	0.212	0.376	0.016	0.021	0.4015
*Callorhinus ursinus*	1.00	0.78	0.39	0.304	0.260	0.024	0.010	0.3703
**Mustelidae**								
*Lutra Canadensis*	2.52	0.82	1.03	0.328	0.166	0.046	0.022	0.9578
*Mustela putorius*	5.88	0.82	2.41	●	0.261	0.112	0.029	2.0761
*Mephitis mephitis*	6.16	0.82	2.53	0.383	0.361	0.085	0.061	2.2300
*Taxidea taxus*	2.80	0.47	0.66	0.732	0.308	0.060	0.053	0.5619
**Canidae**								
*Canis lupus*	6.80	0.82	2.79	0.194	0.219	0.127	0.046	2.3360
*Urocyon cinereoargenteus*	4.42	0.95	2.10	0.146	●	●	0.029	●
*Vulpes vulpes*	4.85	0.82	1.99	0.559	●	●	0.046	●
*Alopex lagopus*	11.3	0.83	4.68	●	0.429	0.079	0.055	4.2204
**Felidae**								
*Felis catus*	4.40	1.60	3.52	0.401	0.193	0.137	0.069	2.8871
*Lynx rufus*	2.64	0.82	1.09	0.168	0.213	0.102	0.052	0.9113
**Artiodactyla**								
**Suidae**								
*Sus scrofa*	5.28	0.82	2.16	0.364	0.184	0.075	0.046	1.9368
*Phacochoerus aethiopicus*	2.79	0.72	1.00	●	0.153	0.074	0.028	0.8913
**Hippopotamidae**								
*Hippopotamus amphibius*	1.00	0.33	0.17	0.165	0.411	0.013	0.005	0.1628
**Bovidae**								
*Aepyceros melampus*	1.00	0.90	0.45	0.357	0.753	0.012	0.020	0.4241
*Kobus ellipsiprymnus*	1.00	1.11	0.56	●	0.488	0.033	0.015	0.5257
*K. kob*	1.00	1.30	0.65	0.534	●	●	0.032	●
*Damaliscus lunatus*	1.00	0.57	0.28	●	●	●	0.056	●
*Syncerus caffer*	1.00	0.40	0.20	0.303	●	●	0.016	●
*Rupicapra rupicapra*	1.00	0.88	0.44	●	0.754	0.010	0.029	0.4057
*Hemitragus jemlahicus*	1.04	●	●	●	0.442	0.026	0.019	●
*Ovis dalli*	1.00	0.75	0.38	●	0.587	0.016	0.015	0.3586
**Moschidae**								
*Moschus berezovskii*	1.41	●	●	●	0.488	0.020	0.014	●
**Cervidae**								
*Cervus elaphus*	1.00	0.82	0.41	0.341	0.602	0.015	0.019	0.3844
*Capreolus capreolus*	2.17	●	●	●	0.389	0.039	0.028	●
*Alces alces*	1.17	0.81	0.47	0.231	0.403	0.031	0.023	0.4314
*Rangifer tarandus*	1.20	0.82	0.49	0.361	0.663	0.010	0.013	0.4721
*Odocoileus hemionus*	1.58	0.94	0.74	0.286	●	●	0.025	●
**Perissodactyla**								
**Equidae**								
*Equus caballus*	1.00	0.49	0.25	●	0.394	0.019	0.010	0.2351
*E. burchelli*	1.00	0.58	0.29	0.535	0.673	0.011	0.010	0.2770
**Proboscidea**								
**Elephantidae**								
*Loxodonta africana*	1.06	0.14	0.08	0.217	0.465	0.004	0.005	0.0750
**Chiroptera**								
**Rhinolophidae**								
*Rhinolophus Ferrumequinum*	1.00	0.82	0.41	●	●	●	0.020	●
**Vespertilionidae**								
*Eptesicus fuscus*	1.64	0.82	0.68	0.529	●	●	0.024	●
*Pipistrellus subflavus*	2.00	0.82	0.82	●	●	●	0.046	●
*P. pipistrellus*	1.26	0.82	0.51	0.621	●	●	0.038	●

*LS, litter size; Lits/yr, litters per year; *b*, annual fecundity; *W_α_*, Adult weight (g); *W*_0_, weight at weaning (g); *δ*, *W*_0_/*W_α_*; *Z*, juvenile mortality (per month); *M*, Adult mortality rate (per month); *S_α_*, survivorship to first parturition; *r*, malthusian parameter or intrinsic population growth rate; ● Data lacking.

### 3.5 The tempo of plateau pika life history

Average fertility *F* = 1.972 (Table 8), and first reproduction ages *α* ≈ 1 year, so *T* = *F*/*α* ≈ 1.972 > 0.60, which means the tempo of plateau pika life history was “fast.”

### 3.6 The dynamics of plateau pika population

**FIGURE 7:**
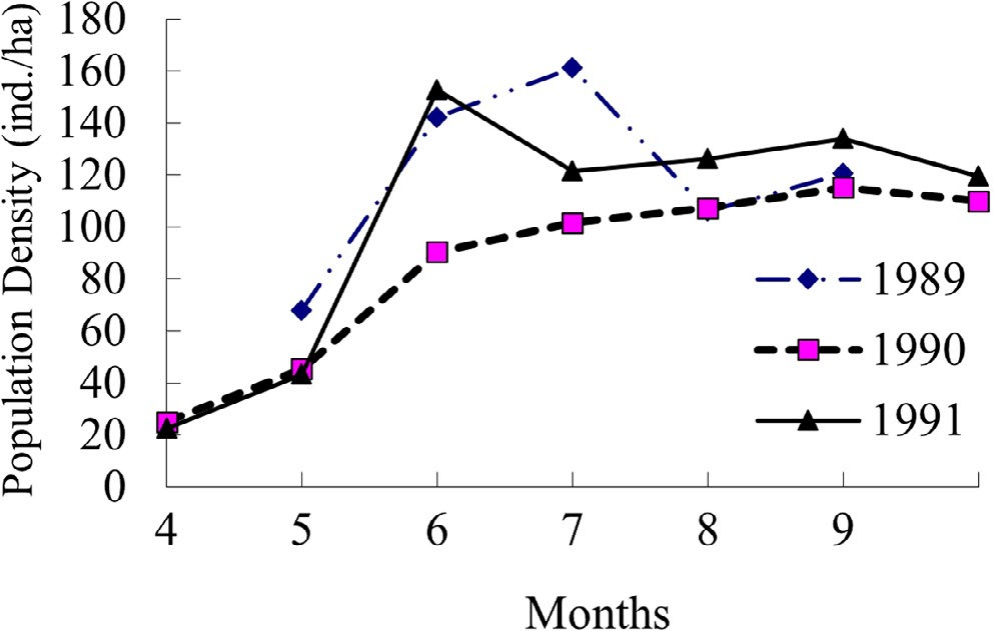
Population dynamics of plateau pika from 1989 to 1991

There were 1,249 individuals marked in the 19 trapping sessions of three survey years, in which 581 individuals were known‐age individuals. Annual variation of the population density was not evident, but the seasonal fluctuation was conspicuous in the 3 years (Figure 7). The dynamics of plateau pika population was coincident with the low intrinsic population growth rate (*r* = .1125), that is, plateau pika population was annually stable.

### 3.7 Age structure and sex ratio of plateau pika population

**FIGURE 8:**
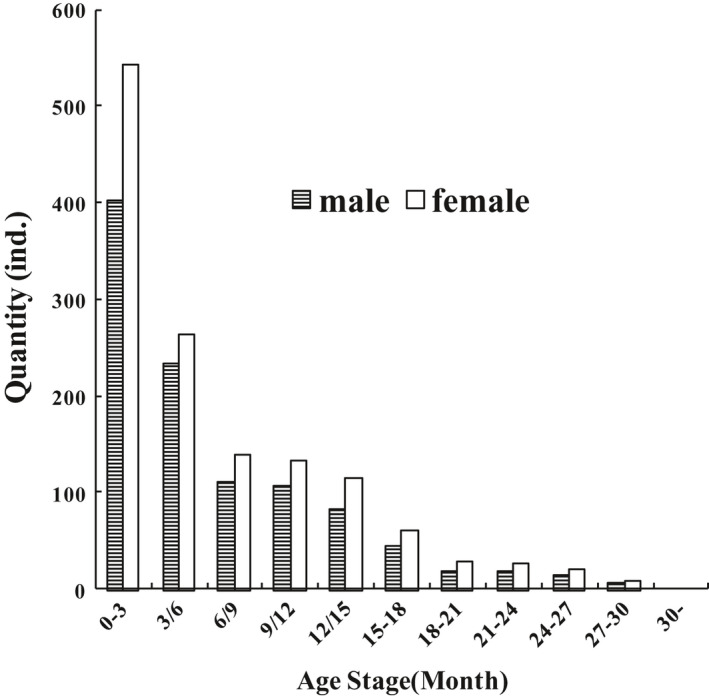
Age structure of plateau pika population

Age structure of the population was pyramidal, that is, juvenile number > subadult number > adult number > old adult number. Female quantity of each age stage was higher than that of the males at the same age stage (Figure 8).

Sex ratio of neonates born in the underground breeding box was 1:1.04. There were 532 females and 405 males captured in the 3 years, and the sex ratio of male/female was 1:1.31.

## 4 DISCUSSION

Conclusions of the experiment are as follows: (1) the fitness index of the plateau pika population was at lower level compared with those of other mammal species; (2) life‐history pattern of plateau pikas was consistent with the style of fast life‐history strategy; (3) the life‐history features of plateau pikas were concordant with the classification standard of Charnov (2002), that is, the ratio of average adult life span to age at first reproduction *E*/*α* ≈ 1.35, the product of reproductive effort and average adult life span *C*·*E* ≈ 1.7, the ratio of the size of an offspring at independence to average adult body mass *I*/*m* ≈ 0.3, which means the size of an offspring at independent age was about 3/10 that of an adult.

The maximum life span of the Haibei alpine meadow population was 931 days, which was 26 days less than that of Qinghai Lake population (Wang & Dai, 1989). Average mortality varied with season, same as Dawu population (Qu et al., 2008).

There were 3 mortality peaks in plateau pika population: juvenile period, breeding period, and the senescent period. The first mortality peak implies that juvenile period is influenced strongly by natural selection; the second mortality peak reflects the cost of reproduction; the third peak implies that the senescence of plateau pika is the result of natural selection (Tables 1 and 2).

Plateau pikas reached body mass max at about 65 days old, which implied that 65 days was the maturity age of plateau pikas (Figure 6). From then on, the food resources obtained from the environment would not be used in the development of organs but be used in the following ways: 1. individual subsistence, that is, sustaining the metabolism of body; 2. breeding posterity.

Correlations among life‐history characteristics have been well studied for many taxa such as mammals, birds, and reptiles (Charnov, 1993). Many life‐history variables are strongly correlated with adult body size (Hanna & Cardillo, 2014; Stearns, 1983; Western, 1979; Wooton, 1987) and can be constrained by phylogeny (Harvey & Pagel, 1991; Stearns, 1983). Other relationships are less intuitive and independent of body size (Charnov, 1993; Gaillard et al.,^1989^; Harvey & Zammuto, 1985; Read & Harvey, 1989). Litter size varied among populations of American pikas independent of differences in body mass (Smith, 1978). Rock‐dwelling pikas (American, collared, many species in Asia) are the same body size as the meadow‐dwelling burrowing pikas—like plateau pikas—yet the two ecotypes of pikas have incredibly different life‐history dynamics (Franken & Hik, 2004).

Mammal life tables can be used in life‐history strategy comparison (Millar & Zammuto, 1983; Promislow & Harvey, 1990; Purvis & Harvey, 1995). Several authors have used their results to categorize mammals on a "fast‐slow" continuum, with species that reproduce early, have large litters, and exhibit short generation times contrasted with those with late maturity, one or fewer offspring per year, and long generation times (Kraus et al., 2005; Oli, 2004; Promislow & Harvey, 1990; Read & Harvey, 1989). Links between elasticity patterns and the fast‐slow continuum may provide a useful tool for classifying mammals according to their likely response to stage‐specific perturbations.

The life‐history tempo of plateau pikas was *T* = *F*/*α* ≈ 1.0972 with average gestation period 18–20 days and life span 490 days, which was consistent with a fast life‐history strategy (Kraus et al., 2005; Oli, 2004).

Reproductive effort is an important factor influencing population fitness. *W*_0_/*W_α_* was calculated as reproductive effort of mammals, that is, average weaning body mass of juveniles divided by average body mass of adults.

### 4.1 Life‐history pattern of the plateau pika (*Ochotona curzoniae*)

#### 4.1.1 Body mass growth mode

Before its 30‐day age, the average body mass of young plateau pikas took a speedy J‐growth. For those aged 30–65 days, the body mass increase speed slowed down and the increase curve took a wide arc form. After the 65‐day age, body mass increase almost stopped (Figure 6). Sexual organs matured at 65‐day age and pikas became fecund, that is, 65 days was the age of sexual maturity for plateau pikas. The females and males were similar morphologically and in size, as other pikas (Smith, 1988).

#### 4.1.2 Survival and death pattern—survival strategy

Most plateau pikas were philopatric and dispersal movements were extremely restricted (Dobson et al., 1998; Qu et al., 2018; Zgurski & Hik, 2012; Zhang et al., 2017); therefore, the survival parameters or mortality parameters were accurate. The overall death pattern of the plateau pikas (*O*. *curzoniae*) was similar to that of other mammals, and the average mortality rate varied with the season (Caughley, 1966). The age‐specific death pattern of the plateau pikas was similar to that of the American pika in the Albanta area (Millar & Zwickel, 1972). However, adult and young mortality rates of the former were both higher than those of the *O*. *princeps*.

There were three mortality peaks for the plateau pika population (Tables 5 and 6). The first was the neonate period, during which only 1/2 of the offspring could survive, which indicated the neonate stage was strongly under natural selection; the second was the reproductive period, during which the pikas paid the price for reproduction; the third was the senility stage, during which the plateau pikas were aging and their vitality was weak. The lowest mortality ages for both females and males were 6–9 months and 21–24 months. For those aged 24–27 months and 27–30 months, the mortality remarkably increased, while individuals aged 30–33 months almost all die (Tables 1 and 2). The drastic mortality increase in the aging stage was the result of natural selection (Emlen, 1970).

Average male mortality was higher than that of female's (${\bar{q}}_{m}$ = 0.43 > ${\bar{q}}_{f}$ = 0.39; Table 3), which might be the result of high agonistic level or high reproduction cost of males' during breeding season (Zhang et al., 2006).

The sexual ratio of the neonates of the plateau pikas (*O*. *curzoniae*) was close to 1. But as the average mortality rate of males was higher than that of females, the sexual ratio in adult population displayed remarkable changes, that is, the female–male ratio = 1.31:1. As a result, the number of survived adult females was higher than that of males, which is identical to the research result by Vessey (1966) of the wild house mouse (*Mus musculus*).

#### 4.1.3 Reproduction Strategy and Mode

Plateau pikas reproduced three times a year (Qu et al., 2008; Wang & Dai, 1989), or ever reproduced 5 times a year (Wang & Smith, 1989), which is different from American pikas (Markham & Whicker, 1973; Millar & Zwickel, 1972; Severaid, 1950).

The average pregnancy period of female plateau pikas is 18–20 days, slightly shorter than that of the American pikas (30 days, Smith, 1978). The average litter size of the former is 4.57, twice as much as that of the latter.

During the field investigation period, plateau pikas (*O*. *curzoniae*) reproduced between April–June, and the time of the first born litter synchronized with the time of plant bourgeon in the alpine meadows, which is the result of long‐term adapting to plateau freezing winter climate. On the one hand, high mortality in the previous winter leads to vast stretches of empty habitats the next spring. On the other hand, during lactation period, females need large amounts of energy (Millar, 1973). After consuming almost all the fat stored in the body, they have to forage more frequently (Sharp, 1973). As a result, they have to wait for the snow‐thawing timing when plants sprout and their food quantity suffices their lactating energy demand (Millar, 1972). Almost all female plateau pikas (*O*. *curzoniae*) got pregnant before plant germination in spring, same as *O*. *princeps* (Krear, 1965; Millar, 1972).

### 4.2 Fitness of plateau pika

The litter size of the plateau pikas (*O*. *curzoniae*) was twice as much as that of the *O*. *princeps*, and both average reproductive rate and survival rate of the subadults and adults of the former were lower than those of the latter. As a result, the fitness level of the former (*r* = .1125) was lower than that of the latter (*r* = 2.172). Among the 46 species of mammals with detailed publicated life tables, the fitness level of *Sylvilagus floridanus* is the highest (6.455), and that of *Moschus berezovskii* is the lowest, followed by that of *Loxodonta Africana* (0.0747). The litter size of *Moschus berezovskii* is bigger than that of *Sylvilagus floridanus*, but annual reproduction time is slightly lower than that of the latter. The average fecundity of the former is almost the same as that of the latter, both at the top level. However, the mortality rates of young and adult *Clethrionmys glareolus* are both 2.5 times as much as those of *Sylvilagus floridanus*. All in all, the fitness level of *Sylvilagus floridanus* population is the highest and it is a rapidly increasing population. The fitness level of *Clethrionmys glareolus* is the lowest and it is a negatively growing population, that is, the population number will decline. The fitness level is similar between plateau pika (*O*. *curzoniae*) and *O*. *princeps*. The effect of survival rate in fitness level in a population is significant.

Populations dynamic trend of plateau pika was concordant with its low *r* value (*r* = .1125), that is, plateau pika populations was rather stable and kept in a rather low population density; therefore, plateau pikas were not harmful to the Haibei alpine meadow ecosystem (Wei et al., 2020).

Population growth rate is a decreasing function of population density for most species, especially in mammals (Tanner, 1966). However, the research result in our investigation is different from that, which may be due to the extremely high mortality rate (91.04%) in winter in the plateau pika population (Wang & Smith, 1988). Restrained by food shortage in the alpine meadow ecosystem in the bitter cold winter, the mortality rate of the plateau pika population is very high in winter and the population can never reach its environmental capacity. The death of the plateau pika population in winter is density‐independent, which brings the population to grow again from a low starting point in the following year.

Food condition is the most important regulatory factor affecting the distribution and the population dynamics of small mammals (Dobson, 1995; Gibert & Krebs, 1981). The *Ochotona* species are nonhibernating species. It is quite difficult for them to find food in the rigid long winter. Food shortage in winter remarkably brings down their ability of resistance to low temperature (Cittadino et al., 1994). *O*. *cansus*, which is distributed in the same region of the plateau pikas (*O*. *curzoniae*), has a habit of storing hay for winter while the plateau pika does not (Su et al., 2004). The status of food in winter affects not only the wintering survival rate of the *Ochotona* but also its reproductive rate, ratio of reproductive individuals, and reproductive period of the following spring (Cole & Batzli, 1978; John, 1986; Taitt & Krebs, 1981). It is a topic worthy of exploration whether the habit of the plateau pikas (*O*. *curzoniae*) not storing hay is a cause of its low fitness level.

In general, the quality of the offspring can be measured by the body mass of the young or the average litter size and body mass (Liu et al., 2002). The average body mass of newborn plateau pikas (*O*. *curzoniae*) is lower than that of newborn *O*. *princeps* (Table 10), which may be a cause of the lower survival rate and hence the lower fitness level of the former.

Reproductive effort is an important parameter estimating population fitness level. Hirshfield and Tinkle (1975) defined reproductive effort as the portion involved in the reproductive procedure of all the energy possessed by an individual, that is, the percentage of the energy consumed by a biont during the reproductive procedure among its total energy. As concrete assessment is difficult by this definition, in general, reproductive effort is estimated based on the ratio between the body mass of the reproductive organ (or tissue) against the body mass (Gadgil & Solbrig, 1972; Tinkle, 1969). However, mammals are taxa that breastfeed and rear the young. After cub birth, the female still keeps making efforts to raise the survival rate of offspring until they wean and become independent. Accordingly, only partial reproductive effort can be estimated based on the ratio of the mass of the reproductive organ against the body mass. It should be more appropriate to take as the index of the reproductive effort of mammals the ratio of the average body mass of the young at weaning (W_0_) against the average body mass of adults (W_α_), or the δ value in Table 10. The δ value of the plateau pikas (*O*. *curzoniae*) is lower than that of the *O*. *princeps* (Table 10), that is, the reproductive effort by plateau pikas (*O*. *curzoniae*) parents is less than that of *O*. *princeps* parents, which may lead to lower quality of the offspring of the former and hence affect their survival rate.

It is an important task for the evolutionary life‐history research to find the trade‐offs between life‐history traits and determine trade‐off degrees between the traits. We analyzed whether trade‐offs exist between life‐history traits such as fecundity and survival rate, residual reproductive value and survival rate, residual reproductive value and fecundity, future fecundity and present fecundity, fecundity and mortality, and reproductive value and mortality rate. However, according to our results, only reproductive value and mortality was significantly negatively correlated with each other; the correlations between all of the other life‐history traits were not significant. There is an unsignificantly positive correlationship between future fecundity and present fecundity and between present fecundity and survival rate. Whether the negative correlation between reproductive value and mortality rate reveals the trade‐offs between the two life‐history traits of the plateau pikas, it is worth further research.

There are approximately 4,200 species of mammals in the world, about 394–544 species are distributed in China (Jiang et al., 2017; Smith & Xie, 2008, 2009; Smith et al., 2018). In the process of our research, detailed life table data of 64 species of 9 orders of mammals have been retrieved, or a mere 65 species of 9 orders including the plateau pika (*O*. *curzoniae*). Among them, the fitness level can be calculated with the life table data of only 46 species of mammals. Due to the incomplete life table data, the fitness value of the other 19 species could not be calculated. Accordingly, it calls for long‐term accumulation and further in‐depth exploration to study the life history and the life‐history strategies of all the species of mammals and to compare their fitness levels. This is an exploration of mammal fitness in population level; however, it must be worth trying some physiological or genetical experiments further to estimate individual fitness and gene fitness.

## CONFLICT OF INTEREST

No conflict of interests.

## AUTHOR CONTRIBUTION

**Haiyan Nie:** Conceptualization (lead); data curation (equal); formal analysis (lead); funding acquisition (equal); investigation (lead); methodology (lead); project administration (equal); resources (equal); software (equal); supervision (equal); validation (equal); visualization (lead); writing – original draft (lead); writing – review and editing (equal).

## Data Availability

The plateau pika life‐history data recorded in this study is available from https://doi.org/10.5061/dryad.rjdfn2zc0 and https://doi.org/10.5061/dryad.gb5mkkwq1.
